# Structures of the first representatives of Pfam family PF06684 (DUF1185) reveal a novel variant of the *Bacillus* chorismate mutase fold and suggest a role in amino-acid metabolism

**DOI:** 10.1107/S1744309109050647

**Published:** 2010-03-05

**Authors:** Constantina Bakolitsa, Abhinav Kumar, Kevin K. Jin, Daniel McMullan, S. Sri Krishna, Mitchell D. Miller, Polat Abdubek, Claire Acosta, Tamara Astakhova, Herbert L. Axelrod, Prasad Burra, Dennis Carlton, Connie Chen, Hsiu-Ju Chiu, Thomas Clayton, Debanu Das, Marc C. Deller, Lian Duan, Ylva Elias, Kyle Ellrott, Dustin Ernst, Carol L. Farr, Julie Feuerhelm, Joanna C. Grant, Anna Grzechnik, Slawomir K. Grzechnik, Gye Won Han, Lukasz Jaroszewski, Hope A. Johnson, Heath E. Klock, Mark W. Knuth, Piotr Kozbial, David Marciano, Andrew T. Morse, Kevin D. Murphy, Edward Nigoghossian, Amanda Nopakun, Linda Okach, Jessica Paulsen, Christina Puckett, Ron Reyes, Christopher L. Rife, Natasha Sefcovic, Henry J. Tien, Christine B. Trame, Christina V. Trout, Henry van den Bedem, Dana Weekes, Aprilfawn White, Qingping Xu, Keith O. Hodgson, John Wooley, Marc-Andre Elsliger, Ashley M. Deacon, Adam Godzik, Scott A. Lesley, Ian A. Wilson

**Affiliations:** a Joint Center for Structural Genomics, http://www.jcsg.org, USA; b Program on Bioinformatics and Systems Biology, Burnham Institute for Medical Research, La Jolla, CA, USA; c Stanford Synchrotron Radiation Lightsource, SLAC National Accelerator Laboratory, Menlo Park, CA, USA; d Protein Sciences Department, Genomics Institute of the Novartis Research Foundation, San Diego, CA, USA; e Center for Research in Biological Systems, University of California, San Diego, La Jolla, CA, USA; f Department of Molecular Biology, The Scripps Research Institute, La Jolla, CA, USA; g University of California, San Diego, La Jolla, CA, USA; h Photon Science, SLAC National Accelerator Laboratory, Menlo Park, CA, USA

**Keywords:** domain of unknown function, structural genomics, chorismate mutase, amino acids, pH-dependent, salt-dependent

## Abstract

The crystal structures of BB2672 and SPO0826 were determined to resolutions of 1.7 and 2.1 Å by single-wavelength anomalous dispersion and multiple-wavelength anomalous dispersion, respectively, using the semi-automated high-throughput pipeline of the Joint Center for Structural Genomics (JCSG) as part of the NIGMS Protein Structure Initiative (PSI). These proteins are the first structural representatives of the PF06684 (DUF1185) Pfam family. Structural analysis revealed that both structures adopt a variant of the *Bacillus* chorismate mutase fold (BCM). The biological unit of both proteins is a hexamer and analysis of homologs indicates that the oligomer interface residues are highly conserved. The conformation of the critical regions for oligomerization appears to be dependent on pH or salt concentration, suggesting that this protein might be subject to environmental regulation. Structural similarities to BCM and genome-context analysis suggest a function in amino-acid synthesis.

## Abstract 2

Structures of the first representatives of PF06684 (DUF1185) reveal a *Bacillus* chorismate mutase-like fold with a potential role in amino-acid synthesis.

## 1. Introduction

To extend the structural coverage of proteins of unknown function, we targeted Pfam protein family PF06684 (domain of unknown function 1185; DUF1185) and determined the crystal structures of two representative members. The *BB2672* gene of *Bordetella bronchiseptica*, which is a causative agent of infectious bronchitis in domestic mammals, encodes a protein with a molecular weight of 20.9 kDa (residues 1–192) and a calculated isoelectric point of 7.1. The *SPO0826* gene of the marine α-proteobacterium *Silicibacter pomeroyi* encodes a protein with a molecular weight of 20.5 kDa (residues 1–­193) and a calculated isoelectric point of 6.2.

Here, we report the crystal structures of BB2672 and SPO0826, which were determined using the semi-automated high-throughput pipeline of the Joint Center for Structural Genomics (JCSG; http://www.jcsg.org; Lesley *et al.*, 2002 ▶) as part of the NIGMS Protein Structure Initiative (PSI). Despite lacking any recognizable sequence similarity to other protein families, both proteins show significant structural similarity to proteins with a *Bacillus* chorismate mutase-like (BCM-like) fold characterized by a β-α-β-α-β-β core that includes a mixed β-sheet (order 1423) with the β4 strand antiparallel to the rest. In bacteria, fungi and higher plants, chorismate mutase (EC 5.4.99.5) catalyzes the isomerization of chorismate to prephenate in the first committed step in the biosynthesis of the aromatic amino acids phenylalanine and tyrosine. While bifunctional chorismate mutases are all-α proteins that form dimers and exhibit feedback inhibition and allostery (Schmidheini *et al.*, 1990 ▶), the mono­functional chorismate mutase adopts a BCM-like fold, is trimeric and follows Michaelis–Menten kinetics. Analysis of the BB2672 crystallo­graphic hexamer reveals a trimer of dimers, with two distinct types of dimerization interface created by an extra N-terminal strand and a β-­hairpin insertion between strands β3 and β4 in the core BCM-like fold. The more extensive interface, which is located at the C-terminus, contains a network of histidine and salt-bridged residues that may indicate environmental regulation of hexamer assembly. The smaller interface at the N-terminus could present a potential ligand-binding site for the DUF1185 family, the genetic context of which supports a role in amino-acid metabolism.

## 2. Materials and methods

### 2.1. Protein production and crystallization

Clones were generated using the Polymerase Incomplete Primer Extension (PIPE) cloning method (Klock *et al.*, 2008 ▶). The genes encoding *B. bronchiseptica* RB50 BB2672 (GenBank NP_889209.1, gi:33601649, Swiss-Prot Q7WJ28) and *S. pomeroyi* DSS-3 SPO0826 (GenBank YP_166079.1, gi:56695728, Swiss-Prot Q5LV76) were amplified by polymerase chain reaction (PCR) from genomic DNA using *PfuTurbo* DNA polymerase (Stratagene) and I-PIPE (Insert) primers (BB2672 forward primer, 5′-ctgtacttccagggcATGTCTT­TGTACGAAATCCGCAAGCGC-3′; BB2672 reverse primer, 5′-­aattaagtcgcgttaGCGCTGGCCGTCGTGCACCGAGACGGC-3′; SPO0826 forward primer, 5′-ctgtacttccagggcATGACCAAGATCC­GCAAGATCGCTG-3′; SPO0826 reverse primer, 5′-aattaagtcgcgtt­aTCTCAGGCCGTCCTTGCCTTCGGCCGCG-3′; target sequences are shown in upper case) that included sequences for the predicted 5′ and 3′ ends. The expression vector pSpeedET, which encodes an amino-terminal tobacco etch virus (TEV) protease-cleavable expression and purification tag (MGSDKIHHHHHHENLYFQ/G), was PCR-amplified with V-PIPE (Vector) primers (forward primer, 5′-taacgcgacttaattaactcgtttaaacggtctccagc-3′; reverse primer, 5′-gcc­ctggaagtacaggttttcgtgatgatgatgatgatg-3′). V-PIPE and I-PIPE PCR products were mixed to anneal the amplified DNA fragments together. *Escherichia coli* GeneHogs (Invitrogen) competent cells were transformed with the V-PIPE/I-PIPE mixture and dispensed onto selective LB–agar plates. The cloning junctions were confirmed by DNA sequencing. Protein expression was performed in selenomethionine-containing medium at 310 K with suppression of normal methionine synthesis. At the end of fermentation, lysozyme was added to the cultures to a final concentration of 250 µg ml^−1^ and the cells were harvested. After one freeze–thaw cycle, the cells were homogenized in lysis buffer [50 m*M* HEPES pH 8.0, 50 m*M* NaCl, 10 m*M* imidazole, 1 m*M* tris(2-carboxyethyl)phosphine hydrochloride (TCEP)] and passed through a Microfluidizer (Microfluidics). The lysates were clarified by centrifugation at 32 500*g* for 30 min and loaded onto nickel-chelating resin (GE Healthcare) pre-equilibrated with lysis buffer; the resin was washed with wash buffer [50 m*M* HEPES pH 8.0, 300 m*M* NaCl, 40 m*M* imidazole, 10%(*v*/*v*) glycerol, 1 m*M* TCEP] and the proteins were eluted with elution buffer [20 m*M* HEPES pH 8.0, 300 m*M* imidazole, 10%(*v*/*v*) glycerol, 1 m*M* TCEP]. The eluates were buffer-exchanged with crystallization buffer (20 m*M* HEPES pH 8.0, 200 m*M* NaCl, 40 m*M* imidazole, 1 m*M* TCEP) using PD-10 columns (GE Healthcare). The BB2672 PD-10 and SPO0826 eluates were treated with 1 mg TEV protease per 15 mg of eluted protein. The digested protein was passed over nickel-chelating resin pre-equilibrated with crystallization buffer and the resin was washed with the same buffer. The flowthrough and wash fractions were combined and concentrated to 20 mg ml^−1^ by centrifugal ultrafiltration (Millipore) for crystallization assays. For SPO0826, TEV cleavage of the expression and purification tag was unsuccessful and the PD-10 eluate was therefore further purified on a HiLoad 16/60 Superdex 200 column (GE Healthcare) using crystallization buffer as the mobile phase. The peak fractions were pooled and concentrated to 15 mg ml^−1^ by centrifugal ultrafiltration for crystallization assays. The two proteins were crystallized by mixing 100 nl protein solution with 100 nl crystallization solution with a 50 µl reservoir volume using the nanodrop vapor-diffusion method (Santarsiero *et al.*, 2002 ▶) with standard JCSG crystallization protocols (Lesley *et al.*, 2002 ▶). Screening for diffraction was carried out using the Stanford Automated Mounting system (SAM; Cohen *et al.*, 2002 ▶) at the Stanford Synchrotron Radiation Lightsource (SSRL, Menlo Park, California, USA). For BB2672, the crystallization reagent consisted of 20%(*w*/*v*) polyethylene glycol 8000 and 0.1 *M* CHES pH 9.5. Ethylene glycol (1,2-ethanediol) was added to a final con­centration of 10%(*v*/*v*) as a cryoprotectant. A plate-shaped crystal of approximate dimensions 0.1 × 0.04 × 0.02 mm was harvested after 26 d at 277 K for data collection. The BB2672 diffraction data were indexed in the orthorhombic space group *C*222_1_ (Table 1 ▶). For SPO0826, the crystallization reagent consisted of 0.1 *M* NaH_2_PO_4_, 0.1 *M* KH_2_PO_4_, 2 *M* NaCl and 0.1 *M* MES pH 6.5. Glycerol was added to a final concentration of 15%(*v*/*v*) as a cryoprotectant. A crystal of approximate dimensions 0.3 × 0.2 × 0.2 mm was harvested after 15 d at 277 K for data collection. The SPO0826 diffraction data were indexed in the tetragonal space group *P*4_3_2_1_2 (Table 2 ▶). The oligomeric states of BB2672 and SPO0826 were determined using a 1 × 30 cm Superdex 200 size-exclusion column (GE Healthcare) coupled with miniDAWN static light-scattering (SEC/SLS) and Optilab differential refractive-index detectors (Wyatt Technology). The mobile phase consisted of 20 m*M* Tris pH 8.0, 150 m*M* NaCl and 0.02%(*w*/*v*) sodium azide. The molecular weight was calculated using *ASTRA* v.5.1.5 software (Wyatt Technology).

### 2.2. Data collection, structure solution and refinement

For BB2672, single-wavelength anomalous diffraction (SAD) data were collected on beamline BL11-1 at SSRL at a wavelength corresponding to the peak of a selenium SAD experiment. The data were collected at 100 K on a MAR Mosaic 325 mm CCD detector using the *Blu-Ice* (McPhillips *et al.*, 2002 ▶) data-collection environment. The SAD data were integrated and reduced using *MOSFLM* (Leslie, 1992 ▶) and scaled with the program *SCALA* (Collaborative Computational Project, Number 4, 1994 ▶). Selenium-substructure solution and phasing were performed with *SHELXD* (Sheldrick, 2008 ▶) and *autoSHARP* (Bricogne *et al.*, 2003 ▶; the mean figure of merit was 0.32 with 25 selenium sites). Automatic model building was performed with *ARP*/*wARP* (Cohen *et al.*, 2004 ▶). Model completion and refinement were performed with *Coot* (Emsley & Cowtan, 2004 ▶) and *REFMAC* v.5.4 (Winn *et al.*, 2003 ▶). The refinement included experimental phase restraints (Pannu *et al.*, 1998 ▶) in the form of Hendrickson–Lattman coefficients from *SHARP* and TLS refinement with one TLS group per chain. Data and refinement statistics for BB2672 are summarized in Table 1 ▶.

For SPO0826, multiple-wavelength anomalous diffraction (MAD) data were collected on beamline BL9-2 at SSRL at wavelengths corresponding to the high-energy remote (λ_1_), inflection (λ_2_) and peak (λ_3_) of a selenium MAD experiment. The data sets were collected at 100 K on a MAR Mosaic 325 mm CCD detector using *Blu-Ice* (McPhillips *et al.*, 2002 ▶). The MAD data were integrated and reduced using *XDS* and scaled with the program *XSCALE* (Kabsch, 1993 ▶). Selenium-substructure solution and phasing were performed with *SHELXD* (Sheldrick, 2008 ▶) and *autoSHARP* (Bricogne *et al.*, 2003 ▶; the mean figure of merit was 0.60 with four selenium sites) and automatic model building was performed with iterative *RESOLVE* (Terwilliger, 2003 ▶). Model completion and refinement were per­formed with *Coot* (Emsley & Cowtan, 2004 ▶) and *REFMAC* v.5.2 (Winn *et al.*, 2003 ▶) using the high-energy remote (λ_1_) data set. The refinement included experimental phase restraints (Pannu *et al.*, 1998 ▶) in the form of Hendrickson–Lattman coefficients from *SHARP* and TLS refinement with one TLS group. Data and refinement statistics for SPO0826 are summarized in Table 2 ▶.

### 2.3. Validation and deposition

**Figure 1:**
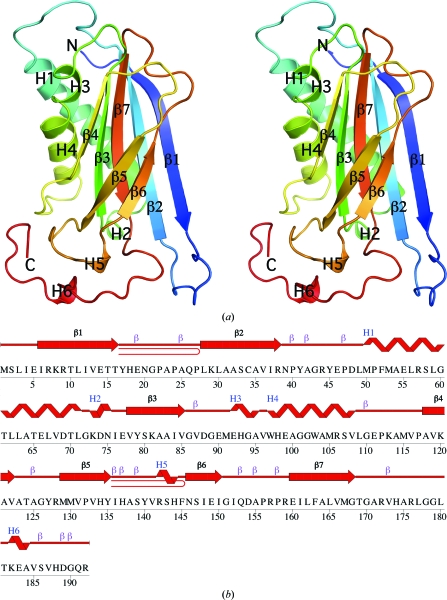
Crystal structure of BB2672 from *B. bronchiseptica*. (*a*) Stereo ribbon diagram of the BB2672 protomer color-coded from the N-terminus (blue) to the C-terminus (red). Helices (H1–H6) and β-strands (β1–β7) are indicated. (*b*) Diagram showing the secondary-structure elements of BB2672 superimposed on its primary sequence in accordance with *PDBsum* (http://www.ebi.ac.uk/pdbsum). For BB2672, the α-helices (H1, H3 and H4), 3_10_-helices (H2, H5 and H6), β-strands (β1–β7) and β-turns (β) are indicated.

The quality of the crystal structure was analyzed using the JCSG Quality Control server (http://smb.slac.stanford.edu/jcsg/QC). This server processes the coordinates and data through a variety of validation tools including *AutoDepInputTool* (Yang *et al.*, 2004 ▶), *MolProbity* (Davis *et al.*, 2007 ▶), *WHAT IF* v.5.0 (Vriend, 1990 ▶), *RESOLVE* (Terwilliger, 2003 ▶) and *MOLEMAN*2 (Kleywegt, 2000 ▶), as well as several in-house scripts, and summarizes the results. Protein quaternary-structure analysis used the *PISA* server (Krissinel & Henrick, 2007 ▶). Fig. 1 ▶(*b*) was adapted from an analysis using *PDBsum* (Laskowski *et al.*, 2005 ▶) and all other figures were prepared with *PyMOL* (DeLano Scientific). Atomic coordinates and experimental structure factors for BB2672 at 1.7 Å resolution and for SP0826 at 2.1 Å resolution have been deposited in the PDB (http://www.pdb.org) and are accessible under codes 3byq and 2qtp, respectively.

## 3. Results and discussion

### 3.1. Overall structure

The crystal structure of BB2672 (Fig. 1 ▶) was determined to 1.7 Å resolution using the SAD method (Table 1 ▶). The final model included three protomers (residues 2–192 of chain *A*, residues 1–192 of chain *B* and residues 2–192 of chain *C*), two tetraethylene glycol (PEG) molecules, 27 ethylene glycol molecules, four chloride ions and 566 water molecules in the asymmetric unit. No electron density was observed for Gly0 (which remained from the N-terminal expression and purification tag after TEV cleavage) in all three chains or for SeMet1 of chains *A* and *C*. Side-chain atoms of Lys73 in chain *A*, SeMet1, Gln26, Lys73 and Asp74 in chain *B*, and Gln26 and Lys73 in chain *C* had poorly defined electron density and were omitted from the model. The Matthews coefficient (*V*_M_; Matthews, 1968 ▶) for BB2672 was 2.3 Å^3^ Da^−1^ and the estimated solvent content was 46.9%. The Ramachandran plot produced by *MolProbity* (Davis *et al.*, 2004 ▶) showed that 97.1% of the residues were in favored regions, with no outliers.

The crystal structure of SPO0826 was determined to 2.1 Å resolution using the MAD method (Table 2 ▶). The final model included one protomer (147 of 194 residues) and 42 water molecules in the asymmetric unit. No electron density was observed for residues from the expression and purification tag, SeMet1–Thr2, Gly103–Ala115, Lys137–His145 or Gly172–Arg193. Side-chain atoms of Lys3, Ala18, Arg20, Arg72, Glu74, Glu77, Glu87, Lys101, Leu116 and Lys122 had poorly defined electron density and were omitted from the model. The Matthews coefficient (*V*_M_; Matthews, 1968 ▶) for SPO0826 was 2.5 Å^3^ Da^−1^ and the estimated solvent content was 51.6%. The Ramachandran plot (Davis *et al.*, 2004 ▶) showed that 95.8% of the residues were in favored regions, with no outliers.

BB2672 forms a single domain composed of seven β-strands (β1–β7; residues 6–16, 28–38, 78–85, 118–122, 129–135, 146–150 and 160–168), three α-helices (H1, H3 and H4; residues 50–71, 92–95 and 97–108) and three 3_10_-helices (H2, H5 and H6; residues 73–75, 142–144 and 182–184) (Fig. 1 ▶). The total β-sheet, α-helical and 3_10_-helical contents were 29.3, 19.9 and 4.7%, respectively. BB2672 contains a central five-stranded mixed β-sheet with 12734 topology that packs against helices H1, H3 and H4. Two additional strands (β5–β6) flank the sheet from the opposite side.

SPO0826 also forms a single domain composed of seven β-strands (β1–β7; residues 4–17, 20–36, 78–85, 119–123, 130–134, 147–151 and 161–170), but with only two α-helices (H1 and H3; residues 48–69 and 91–97) and one 3_10_-helix (H2; residues 73–75). The total β-sheet, α-­helical and 3_10_-helical contents were 43.5, 19.7 and 2.0%, respectively.

### 3.2. Similarity between BB2672 and SPO0826

**Figure 2:**
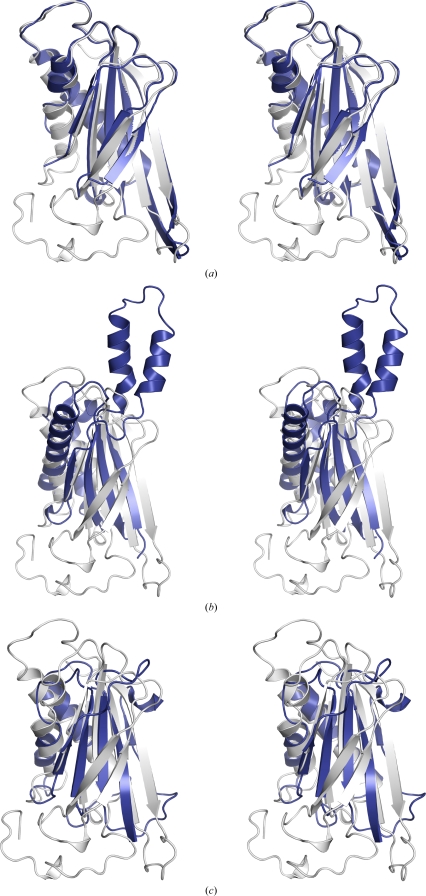
Stereo ribbon diagrams comparing BB2672 with other related homologs. (*a*) Superposition of BB2672 (gray; PDB code 3byq) and SPO0826, a DUF1185 homolog from *S. pomeroyi* (blue; PDB code 2qtp). (*b*) Superposition of BB2672 (gray) with the OmpA-like domain of RmpM from *N. meningitidis* (blue; PDB code 1r1m) and (*c*) with the monofunctional chorismate mutase from *B. subtilis* (blue; PDB code 2cht).

BB2672 and SPO0826 are closely homologous, with a sequence identity of 39%, and share the same overall fold and tertiary structure. Superposition of the two structures extends over 142 equivalent C^α^ atoms, with an r.m.s.d. of 1.4 Å (Fig. 2 ▶ *a*). The dis­ordered residues in the SPO0826 structure correspond to a large C-­terminal region of BB2672 that encompasses helix H4 (residues 97–109), most of the H4–β4 loop (residues 110–115), the β5–β6 loop (residues 137–145), including the 3_10_-helix H5, and the C-terminal region (residues 172–193) after β7, including 3_10_-helix H6. In BB2672, consecutive glycines (residues 101–102) located after the first turn of helix H4 (Fig. 1 ▶ *b*) result in a break in the main-chain hydrogen bonding of the helix, indicating a degree of structural plasticity in this region. Gly102 is highly conserved among BB2672 homologs, suggesting that the conformational flexibility in H4 might have functional implications. The region C-terminal of β7, including 3_10_-­helix H6, has high *B* values but is stabilized through interactions with the β5–β6 loop that appear to be critical for formation of the hexamer.

### 3.3. Similarity to other proteins

BB2672 and SPO0826 both show significant structural similarity to proteins with the *Bacillus* chorismate mutase-like (BCM-like) fold (http://scop.mrc-lmb.cam.ac.uk/scop/data/scop.b.e.bie.j.b.b.html), a ‘circular permutation’ variant of the thioredoxin-like fold (Qi & Grishin, 2005 ▶). A *FATCAT* (Ye & Godzik, 2004 ▶) database search identified several proteins with this fold that had significant structural similarities to BB2672. Among these, the top hit was the C-­terminal domain of RmpM, an outer mem­brane protein from *Neisseria meningitidis* (PDB code 1r1m; Grizot & Buchanan, 2004 ▶), with a C^α^ r.m.s.d. of 3.1 Å and 7% sequence identity over 96 residues (Fig. 2 ▶ *b*). Superposition with the monofunctional chorismate mutase from *Bacillus subtilis* (PDB code 2cht; Chook *et al.*, 1993 ▶) resulted in similar r.m.s.d. values (2.5 Å with 10% sequence identity over 86 residues; Fig. 2 ▶ *c*). In addition to the core BCM-like fold, BB2672 contains an extra N-terminal strand (β1) and a β-­hairpin (strands β5–β6) inserted between the third and fourth strands of the BCM β-sheet (Fig. 2 ▶ *c*). In RmpM, BB2672 strands β5–β6 are replaced by two helices that extend out in the opposite direction from the β-strands (Fig. 2 ▶ *b*), while the region C-terminal of BB2672 strand β7 is absent from both structures (Figs. 2 ▶ *b* and 2 ▶ *c*).

**Figure 3:**
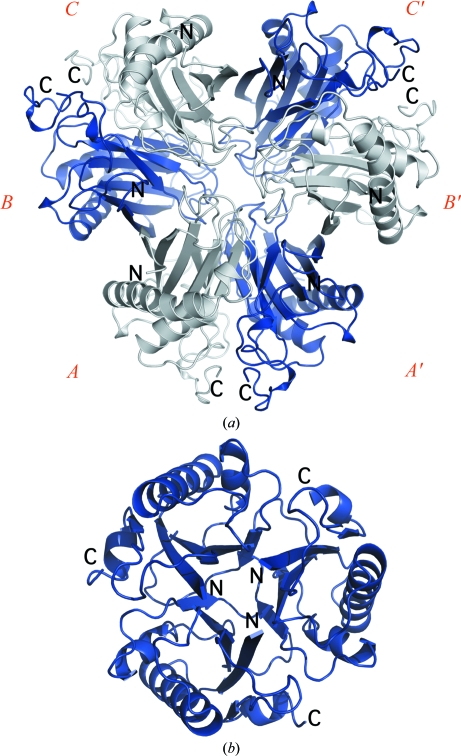
Oligomerization states of BB2672 and monofunctional BCM. (*a*) Ribbon diagram of the BB2672 hexamer showing the arrangement of consecutive subunits (top view). The three molecules present in the asymmetric unit are labeled *A*, *B* and *C* in red. *A*′, *B*′ and *C*′ are the corresponding crystallographically related molecules that together form the BB2672 hexamer. Protomers with their N-terminus pointing to the front are shown in gray and those with their N-terminus pointing towards the back are shown in blue. The C-terminal ‘dimer’ interface occurs between protomers *B*–*C*, *B*′–*C*′ and *A*–*A*′. The N-terminal interface occurs between protomers *A*–*B*, *A*′–*B*′ and *C*–*C*′. (*b*) Ribbon diagram of the BCM trimer (PDB code 2cht, top view) showing the parallel arrangement of protomers resulting in three equivalent interfaces.

Analysis of BB2672 using the *PISA* server (Krissinel & Henrick, 2007 ▶) indicated that a hexamer was the likely quaternary form. Analyt­ical size-exclusion chromatography in combination with static light scattering indicated that both BB2672 and SPO0826 were hexamers in solution. The BB2672 hexamer corresponds to a ‘trimer of dimers’ with approximate dimensions of 50 × 70 × 80 Å, with the helices positioned on the outside and the β-strands forming the inner core (Fig. 3 ▶ *a*). This arrangement creates two main types of interface. The interface between chains *B* and *C* (and equivalents between *A* and *A*′ and between *C*′ and *B*′; Fig. 3 ▶ *a*) is extensive, burying a surface area of 1960 Å^2^ per protomer with 32 hydrogen bonds and six salt bridges. This interface involves strands β4–β6 and helix H5 from both protomers, and the first turn of helix H4, loops β2–H1 and β3–H3 from one protomer that contacts the region C-terminal of β7 in the other protomer. Two strictly conserved pairs of salt bridges (Glu90–Arg176 and Glu92–Arg172) account for the majority of the electrostatic interactions along this interface. Both sets of salt bridges are almost entirely shielded from solvent upon oligomerization. Residues in the first half-turn of helix H3, loop β2–H1, loop β3–H3 and the region C-­terminal of β7 are also highly conserved, suggesting that this interface serves a conserved functional purpose. Analysis of the BB2672 hexamer structure indicates that this ‘C-terminal’ or ‘dimer’ interface may provide dimers as the initial oligomerization building blocks which then assemble to form the hexamer. However, this interface was not observed in the SPO0826 structure, which crystallized with a single molecule in the asymmetric unit.

The second interface, which is observed in both the BB2672 and the SPO0826 structures, is formed between chains *A* and *B* (and equivalently between *C* and *C*′ and between *B*′ and *A*′; Fig. 3 ▶ *a*). This ‘N-terminal’ interface mainly involves interaction of the β1 strands of adjacent chains in an antiparallel manner to form a continuous β-­sheet across interacting protomers. In BB2672 this ,interface buries a surface area of 950 Å^2^ per protomer with 18 hydrogen bonds and four salt bridges. Pairs of intermolecular salt bridges (Lys8–Glu14 and Glu19–Arg7) anchor the adjacent ends of the corresponding β1 strands. With the exception of the strictly conserved Arg7–Glu19 salt bridge, no other highly conserved intermolecular interactions are observed in this interface. In BB2672, the N-terminal interface is involved in the formation of the hexamer, while in SPO0826, it is only involved in crystal contacts. This interface is not predicted by *PISA* (Krissinel & Henrick, 2007 ▶) to form a stable dimer and thus seems to only be stable when the molecules assemble as a hexamer. Both the β1 strand and the region C-terminal of strand β7 are absent in classic BCM folds, where the subunits are arranged in parallel to form homotrimers (Chook *et al.*, 1993 ▶; Fig. 3 ▶ *b*). Thus, the two distinct types of interface observed in the BB2672 hexamer could result from these two novel additions to the classic BCM-like fold.

**Figure 4:**
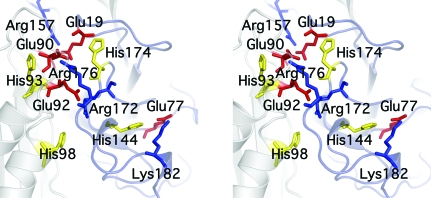
Stereo close-up view of the cavity in the BB2672 C-terminal ‘dimer’ interface. Conserved histidines and salt bridges are indicated.

Analysis of the BB2672 hexamer structure using the *CastP* server (Binkowski *et al.*, 2003 ▶) revealed a large cavity (∼1000 Å^3^) located along the C-terminal or dimer interface and including residues from helix H4, the H4–β4 loop, β5, the β5–H5 loop, the 3_10_-helix H5 and the region C-terminal of β7. Proximal to this cavity, a highly con­served cluster of four histidines, His93, His98, His144, His174, is located within hydrogen-bonding distance of residues involved in the formation of intermolecular salt bridges (Fig. 4 ▶). All four histidines are located in potentially flexible regions of the molecule: His144 and His174 in loops and His87 and His98 at the beginning of helices H3 and H4, respectively. Helix H3 is a very short 3_10_-helix, whereas His98 is located on a turn before a break in the main-chain hydrogen-bonding pattern of helix H4. In SPO0826, most of these regions are disordered, indicating flexibility. This clustering of histidine residues in regions of some conformational variability and the proximity of salt bridges involved in oligomerization suggests that assembly of the BB2672 hexamer could be modulated by pH or salt concentration. The imidazole of the strictly conserved His144 is the only solvent-exposed side chain of these His residues in the structure, suggesting that it is the less solvent-accessible histidines that are likely to control pH-dependent oligomerization. The observation that SPO0826 crystallizes as a monomer in high salt and acidic pH (2 *M* NaCl pH 6.5), while demonstrating the same hexameric oligomerization state in solution as BB2672 under alkaline and low-salt conditions (150 m*M* NaCl pH 8.0), lends further support to the hypothesis that pH or salt concentration might be a key factor in oligomerization.

RmpM, the most similar structure to BB2672, is a putative peptido­glycan-binding protein that interacts with integral outer membrane proteins such as porins and with transporters implicated in iron acquisition and bacterial pathogenesis (Grizot & Buchanan, 2004 ▶). The peptidoglycan-binding site is located along a highly conserved hydrophilic groove that partially overlaps with a cavity containing highly conserved residues along the C-terminal dimerization interface of BB2672 (His93, Arg176 and Glu90; Grizot & Buchanan, 2004 ▶). However, structural alignment shows no sequence similarity between BB2672 and the proposed binding residues in RmpM, and access to the cavity in BB2672 is partially occluded by the β2–H1 and β3–H3 loops.

In the monofunctional chorismate mutase from *Bacillus subtilis* (PDB code 2cht), the active sites are located at each of the three subunit interfaces and the reaction involves stabilization of the charged transition state of chorismate *via* the formation of ionic bonds with a number of acidic and basic side chains in BCM (Chook *et al.*, 1993 ▶). A number of studies have established that these electro­static effects provide the main drive in catalysis (Kast *et al.*, 1996 ▶). Electrostatic catalysis can additionally be mediated by metal ions and histidine proton shuttling (Christianson & Cox, 1999 ▶). Although the C-terminal interface in BB2672 displays conservation of an electrostatic surface analogous to monofunctional BCM, the lack of sequence similarity between the two proteins, as well as their different oligomerization states, makes it unlikely that they share a common ligand. Assuming that the C-terminal interface plays a role in environmental regulation of the BB2672 hexamer assembly, ligand binding might occur along the less extensive and presumably less stable N-­terminal interface that is likely not formed prior to BB2672 hexamerization.

Several genes predicted (http://string.embl.de) to have functional associations with BB2672 include putative exported proteins (BB2673, BB0971 and BB4692), transcriptional regulators implicated in pathogenesis and multiple antibiotic resistance (BB2675 and BB1771) and various proteins (BB1772, BB1773 and BB1774) involved in branched-chain amino-acid (BCAA; *i.e.* leucine, isoleucine and valine) metabolism. A similar pattern is observed in the genomic neighborhood of SPO0826, with several genes (SPO0822, SPO0823, SPO0824 and SPO0825) involved in ATP-binding cassette (ABC) type BCAA transport and this pattern is repeated again in other BB2672 orthologs, lending further support to an association of this family with amino-acid metabolism.

The BB2672 protein family (DUF1185; PF06684) contains more than 200 sequence homologs, all of which are approximately 180 residues in length; they are mostly found in proteobacteria, but also in firmicutes and uncultured bacteria from ocean, soil and human microbiota. Members of the DUF1185 family are also present in several human and animal pathogens from burkholderia and bordetella, as well as clostridia, where their genome location on virulence islands suggests a possible role in pathogenesis (Nierman *et al.*, 2004 ▶). In some pathogenic bacteria, one of the modes of BCAA transportation is thought to be sensitive to stress induced by changes in osmolarity, pH or temperature (Vijaranakul *et al.*, 1998 ▶). In this context, BB2672 and its homologs might present attractive drug targets since, similar to chorismate mutases, DUF1185 homologs are not found in mammals.

## 4. Conclusion

The structural similarities between BB2672 and monofunctional BCM, as well the genomic neighborhood of BB2672 and homologs, appear to suggest involvement of the DUF1185 (PF06684) family in amino-acid metabolism. However, the presence of additional secondary-structure elements in BB2672 and differences in oligomerization states (hexamer for BB2672, trimer for BCM) and the lack of detectable sequence similarity between BB2672 and BCM make it unlikely that BB2672 and BCM share the same ligands. Finally, comparison of the BB2672 and SPO0826 structures led us to propose that oligomerization and potentially function in the DUF1185 family could be subject to pH or salt regulation. Thus, BB2672 homologs may also act as environmental sensors, enabling bacterial adaptation and survival under a range of conditions.

The availability of more DUF1185 sequences and structures should shed light on the evolutionary history of this intriguing protein family. The information presented here, in combination with further bio­chemical and biophysical studies, should yield valuable insights into the functional role of BB2672. Models for BB2672 homologs can be accessed at http://www1.jcsg.org/cgi-bin/models/get_mor.pl?key=3byqA. Additional information about BB2672 and SPO0826 is available from TOPSAN (Krishna *et al.*, 2010 ▶) at http://www.topsan.org/explore?PDBid=3byq and http://www.topsan.org/explore?PDBid=2qtp, respectively.

## Supplementary Material

PDB reference: BB2672 from *B. bronchi­septica*, 3byq, r3byqsf

PDB reference: SPO0826 from *S. pomeroyi*, 2qtp, r2qtpsf

## Figures and Tables

**Table 1:** Summary of crystal parameters, data-collection and refinement statistics for BB2672 (PDB code 3byq) Values in parentheses are for the highest resolution shell.

	λ_1_ SAD-Se
Space group	*C*222_1_
Unit-cell parameters (Å)	*a* = 96.54, *b* = 133.13, *c* = 92.54
Data collection	
Wavelength (Å)	0.9791
Resolution range (Å)	29.9–1.70 (1.74–1.70)
No. of observations	323104
No. of unique reflections	65104
Completeness (%)	99.2 (96.6)
Mean *I*/σ(*I*)	11.7 (2.0)
*R*_merge_ on *I*† (%)	10.7 (71.6)
Model and refinement statistics	
Resolution range (Å)	29.9–1.70
No. of reflections (total)	65081‡
No. of reflections (test)	3297
Completeness (%)	99.0
Data set used in refinement	λ_1_ SAD-Se
Cutoff criterion	|*F*| > 0
*R*_cryst_§	0.138
*R*_free_¶	0.169
Stereochemical parameters	
Restraints (r.m.s.d. observed)	
Bond lengths (Å)	0.015
Bond angles (°)	1.64
Average isotropic *B* value (Å^2^)	21.7††
ESU‡‡ based on *R*_free_ value (Å)	0.08
Protein residues/atoms	574/4454
Water molecules/solvent/ions	566/29/4

† *R*_merge_ = 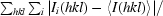 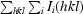.

‡ The number of unique reflections used in refinement is typically slightly less than the total number that were integrated and scaled. Reflections are excluded owing to systematic absences, negative intensities and rounding errors in the resolution limits and unit-cell parameters.

§ *R*_cryst_ = 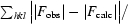 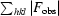, where *F*_calc_ and *F*_obs_ are the calculated and observed structure-factor amplitudes, respectively.

¶ *R*_free_ is the same as *R*_cryst_ but for 5.1% of the total reflections chosen at random and omitted from refinement.

†† This value represents the total *B* that includes TLS and residual *B* components.

‡‡ Estimated overall coordinate error (Collaborative Computational Project, Number 4, 1994 ▶; Cruickshank, 1999 ▶).

**Table 2:** Summary of crystal parameters, data-collection and refinement statistics for SPO0826 (PDB code 2qtp) Values in parentheses are for the highest resolution shell.

	λ_1_ MAD-Se	λ_2_ MAD-Se	λ_3_ MAD-Se
Space group	*P*4_3_2_1_2		
Unit-cell parameters (Å)	*a* = *b* = 94.73, *c* = 47.15		
Data collection			
Wavelength (Å)	0.9116	0.9792	0.9791
Resolution range (Å)	27.3–2.10 (2.15–2.10)	27.3–2.10 (2.15–2.10)	27.3–2.10 (2.15–2.10)
No. of observations	91717	91261	91289
No. of unique reflections	13007	13003	13021
Completeness (%)	99.7 (98.6)	99.6 (98.3)	99.7 (99.7)
Mean *I*/σ(*I*)	20.6 (2.7)	17.0 (2.6)	17.5 (2.5)
*R*_merge_ on *I*† (%)	4.8 (79.5)	6.2 (87.5)	6.2 (89.1)
Model and refinement statistics			
Resolution range (Å)	27.3–2.10		
No. of reflections (total)	12970‡		
No. of reflections (test)	631		
Completeness (%)	99.5		
Data set used in refinement	λ_1_ MAD-Se		
Cutoff criterion	|*F*| > 0		
*R*_cryst_§	0.204		
*R*_free_[Table-fn tfn10]	0.259		
Stereochemical parameters			
Restraints (r.m.s.d. observed)			
Bond lengths (Å)	0.017		
Bond angles (°)	1.68		
Average isotropic *B* value (Å^2^)	60.2[Table-fn tfn11]		
ESU[Table-fn tfn12] based on *R*_free_ value (Å)	0.17		
Protein residues/atoms	147/1103		
Water molecules	42		

† *R*_merge_ = 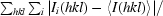 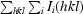.

‡ The number of unique reflections used in refinement is typically slightly less than the total number that were integrated and scaled. Reflections are excluded owing to systematic absences, negative intensities and rounding errors in the resolution limits and unit-cell parameters.

§ *R*_cryst_ = 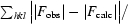 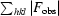, where *F*_calc_ and *F*_obs_ are the calculated and observed structure-factor amplitudes, respectively.

¶ *R*_free_ is the same as *R*_cryst_ but for 4.9% of the total reflections chosen at random and omitted from refinement.

†† This value represents the total *B* that includes TLS and residual *B* components.

‡‡ Estimated overall coordinate error (Collaborative Computational Project, Number 4, 1994 ▶; Cruickshank, 1999 ▶).

## References

[bb1] Binkowski, T. A., Naghibzadeh, S. & Liang, J. (2003). *Nucleic Acids Res.* **31**, 3352–3355. 10.1093/nar/gkg512 PMC168919 12824325

[bb2] Bricogne, G., Vonrhein, C., Flensburg, C., Schiltz, M. & Paciorek, W. (2003). *Acta Cryst.* D**59**, 2023–2030. 10.1107/s0907444903017694 14573958

[bb3] Chook, Y. M., Ke, H. & Lipscomb, W. N. (1993). *Proc. Natl Acad. Sci. USA*, **90**, 8600–8603. 10.1073/pnas.90.18.8600 PMC47405 8378335

[bb4] Christianson, D. W. & Cox, J. D. (1999). *Annu. Rev. Biochem.* **68**, 33–57. 10.1146/annurev.biochem.68.1.33 10872443

[bb5] Cohen, A. E., Ellis, P. J., Miller, M. D., Deacon, A. M. & Phizackerley, R. P. (2002). *J. Appl. Cryst.* **35**, 720–726. 10.1107/s0021889802016709 PMC4041710 24899734

[bb6] Cohen, S. X., Morris, R. J., Fernandez, F. J., Ben Jelloul, M., Kakaris, M., Parthasarathy, V., Lamzin, V. S., Kleywegt, G. J. & Perrakis, A. (2004). *Acta Cryst.* D**60**, 2222–2229. 10.1107/S0907444904027556 15572775

[bb7] Collaborative Computational Project, Number 4 (1994). *Acta Cryst.* D**50**, 760–763.

[bb8] Cruickshank, D. W. J. (1999). *Acta Cryst.* D**55**, 583–601. 10.1107/s0907444998012645 10089455

[bb9] Davis, I. W., Leaver-Fay, A., Chen, V. B., Block, J. N., Kapral, G. J., Wang, X., Murray, L. W., Arendall, W. B. III, Snoeyink, J., Richardson, J. S. & Richardson, D. C. (2007). *Nucleic Acids Res.* **35**, W375–W383. 10.1093/nar/gkm216 PMC1933162 17452350

[bb10] Davis, I. W., Murray, L. W., Richardson, J. S. & Richardson, D. C. (2004). *Nucleic Acids Res.* **32**, W615–W619. 10.1093/nar/gkh398 PMC441536 15215462

[bb11] Emsley, P. & Cowtan, K. (2004). *Acta Cryst.* D**60**, 2126–2132. 10.1107/S0907444904019158 15572765

[bb12] Grizot, S. & Buchanan, S. K. (2004). *Mol. Microbiol.* **51**, 1027–1037. 10.1111/j.1365-2958.2003.03903.x 14763978

[bb13] Kabsch, W. (1993). *J. Appl. Cryst.* **26**, 795–800.

[bb14] Kast, P., Asif-Ullah, M., Jiang, N. & Hilvert, D. (1996). *Proc. Natl Acad. Sci. USA*, **93**, 5043–5048. 10.1073/pnas.93.10.5043 PMC39403 8643526

[bb15] Kleywegt, G. J. (2000). *Acta Cryst.* D**56**, 249–265. 10.1107/s0907444999016364 10713511

[bb16] Klock, H. E., Koesema, E. J., Knuth, M. W. & Lesley, S. A. (2008). *Proteins*, **71**, 982–994. 10.1002/prot.21786 18004753

[bb17] Krishna, S. S., Weekes, D., Bakolitsa, C., Elsliger, M.-A., Wilson, I. A., Godzik, A. & Wooley, J. (2010). *Acta Cryst.* F**66**, 1143–1147. 10.1107/S1744309110035736 PMC2954197 20944203

[bb18] Krissinel, E. & Henrick, K. (2007). *J. Mol. Biol.* **372**, 774–797. 10.1016/j.jmb.2007.05.022 17681537

[bb19] Laskowski, R. A., Chistyakov, V. V. & Thornton, J. M. (2005). *Nucleic Acids Res.* **33**, D266–D268. 10.1093/nar/gki001 PMC539955 15608193

[bb20] Lesley, S. A. *et al.* (2002). *Proc. Natl Acad. Sci. USA*, **99**, 11664–11669.

[bb21] Leslie, A. G. W. (1992). *Jnt CCP4/ESF–EACBM Newsl. Protein Crystallogr.* **26**

[bb22] Matthews, B. W. (1968). *J. Mol. Biol.* **33**, 491–497. 10.1016/0022-2836(68)90205-2 5700707

[bb23] McPhillips, T. M., McPhillips, S. E., Chiu, H.-J., Cohen, A. E., Deacon, A. M., Ellis, P. J., Garman, E., Gonzalez, A., Sauter, N. K., Phizackerley, R. P., Soltis, S. M. & Kuhn, P. (2002). *J. Synchrotron Rad.* **9**, 401–406. 10.1107/s0909049502015170 12409628

[bb24] Nierman, W. C. *et al.* (2004). *Proc. Natl Acad. Sci. USA*, **101**, 14246–14251.

[bb25] Pannu, N. S., Murshudov, G. N., Dodson, E. J. & Read, R. J. (1998). *Acta Cryst.* D**54**, 1285–1294. 10.1107/s0907444998004119 10089505

[bb26] Qi, Y. & Grishin, N. V. (2005). *Proteins*, **58**, 376–388. 10.1002/prot.20329 15558583

[bb27] Santarsiero, B. D., Yegian, D. T., Lee, C. C., Spraggon, G., Gu, J., Scheibe, D., Uber, D. C., Cornell, E. W., Nordmeyer, R. A., Kolbe, W. F., Jin, J., Jones, A. L., Jaklevic, J. M., Schultz, P. G. & Stevens, R. C. (2002). *J. Appl. Cryst.* **35**, 278–281.

[bb28] Schmidheini, T., Mosch, H. U., Evans, J. N. & Braus, G. (1990). *Biochemistry*, **29**, 3660–3668. 10.1021/bi00467a011 2187528

[bb29] Sheldrick, G. M. (2008). *Acta Cryst.* A**64**, 112–122. 10.1107/S0108767307043930 18156677

[bb30] Terwilliger, T. C. (2003). *Acta Cryst.* D**59**, 1174–1182. 10.1107/S0907444903009922 PMC2745880 12832760

[bb31] Vijaranakul, U., Xiong, A., Lockwood, K. & Jayaswal, R. K. (1998). *Appl. Environ. Microbiol.* **64**, 763–767. 10.1128/aem.64.2.763-767.1998 PMC106115 9464420

[bb32] Vriend, G. (1990). *J. Mol. Graph.* **8**, 52–56. 10.1016/0263-7855(90)80070-v 2268628

[bb34] Winn, M. D., Murshudov, G. N. & Papiz, M. Z. (2003). *Methods Enzymol.* **374**, 300–321. 10.1016/S0076-6879(03)74014-2 14696379

[bb35] Yang, H., Guranovic, V., Dutta, S., Feng, Z., Berman, H. M. & Westbrook, J. D. (2004). *Acta Cryst.* D**60**, 1833–1839. 10.1107/S0907444904019419 15388930

[bb36] Ye, Y. & Godzik, A. (2004). *Nucleic Acids Res.* **32**, W582–W585. 10.1093/nar/gkh430 PMC441568 15215455

